# 575 Are There Racial Disparities in Burn Center Care?

**DOI:** 10.1093/jbcr/irac012.203

**Published:** 2022-03-23

**Authors:** Palmer Q Bessey, Jamie M Heffernan, Jim Gallagher

**Affiliations:** New York-Presbyterian Hospital / Weill Cornell Medicine, New York, New York; NY Presbyterian Weill Cornell, New York, New York; none, Phoenicia, New York

## Abstract

**Introduction:**

Events in the past two years highlighted disparities in outcomes based on race for several disease conditions. There have been reports suggesting racial disparities may exist for burn care. This burn center has long been committed to serving all groups within the city. The purpose of this study was to determine if race might influence the outcome of burn care at this center.

**Methods:**

We reviewed inpatient admissions for eight years, 2012 through 2019. Racial identity was reported by patients and their families and categorized as Asian, Black, Latino, or White. Place of residence was categorized by Zip code. Other variables were recorded. The revised Baux Score (RBS) was calculated according to Osler and Hosmer (2010). Several logistic regression models were tested for each racial group. They related death to RBS as well to age, burn size, and inhalation injury. An additional predictor variable for race was then added to each regression model. Statistical calculations were performed using SAS 9.4 (SAS Institute Inc., Cary, NC).

**Results:**

5,440 patients were admitted as inpatients. Of these 4,626 (85%) resided in a zip code within this city, and patients lived in essentially every zip code. The racial distribution of the patients reflected the racial distribution of the city and of the several neighborhoods within the city. Death was strongly related to RBS or to age, burns size, and inhalation injury for all groups. In all cases, an additional predictor for race was not significant.

**Conclusions:**

This burn center serves all areas and populations of the city. Outcome of burn center care was related to burn extent, age, inhalation injury. There were no detectable differences in the outcome of burn center care associated with race. Burn Center care should be equally effective for all races.

